# Making the health system work by and for Indigenous women in Guatemala: a community led multisectoral collaboration

**DOI:** 10.1136/bmj.k4677

**Published:** 2018-12-07

**Authors:** Claudia Nieves Velásquez, Maria del Rosario Garcia Meza, Daria Ukhova, Silvia Xinico, Susana Palma, Sarah Simpson

**Affiliations:** 1 Guatemala City, Guatemala; 2 Berlin, Germany; 3 Alianza Nacional de Organizaciones de Mujeres Indigenas por la Salud Reproductiva Nutrición y Educación (ALIANMISAR), Guatemala City, Guatemala; 4 USAID Health Education and Policy Project, Guatemala City, Guatemala; 5 EquiACT, Lyon, France

## Abstract

Claudia Nieves Velásquez and colleagues report how a community led national alliance of Indigenous women’s organisations is working to improve the delivery of healthcare for Indigenous women through collaboration with other community based organisations, government (health and ombudsman), and international partners

Inequities in indigenous peoples’ health persist, reflecting the continued disadvantage and discriminatory attitudes experienced by indigenous people worldwide that affect their use of health services.^1^ ^2^ For Guatemala, where nearly half of the population is indigenous—mainly Mayan groups—inequities remain a persistent challenge. Most of the country’s Indigenous peoples have higher rates of poverty and were profoundly affected by the civil war (1960-96), with about 83% of the two million victims belonging to one of the Mayan Indigenous groups.^1^ ^3^ ^4^ ^5^ ^6^ The Alianza Nacional de Organizaciones de Mujeres Indigenas por la Salud Reproductiva Nutrición y Educación (National Alliance of Indigenous Women’s Organizations for Reproductive Health, Nutrition, and Education, ALIANMISAR) is one example of how Guatemala’s Indigenous communities are working to deal with these challenges. ALIANMISAR’s efforts built on the 1996 Peace Accords, which marked the end of the civil war and emphasised the need for civil society stewardship and active involvement in governance.^7^ The accords also mandated a 50% increase in the public health budget, focused on preventing ill health, decreasing maternal and infant mortality, and eradicating polio and measles.

ALIANMISAR is a network led by Indigenous women and was formed in 2006 to improve the quality and cultural acceptability of healthcare provided to Indigenous women.^5^ As part of its mission, ALIANMISAR monitors a range of public health services at national, departmental, and municipal levels, in collaboration with other community based organisations, the executive and legislative sectors of the government (the Ministry of Health and the Ombudsman for Human Rights), and international partners (see suppl 1 on bmj.com). Monitoring of health services by ALIANMISAR volunteers and staff from the ombudsman’s field offices includes interviews with service providers and users and an inspection of the facilities, equipment, supplies and medicines. To date, joint monitoring has contributed to important improvements in health policy and legislation, health services, and infrastructure for Indigenous women.

We focus on the factors that have enabled this multisectoral collaboration; impetus for this analysis comes from recognition that multisectoral collaboration is essential to achieve the sustainable development goals.^8^

## Why was monitoring needed?

After more than a decade of post-war reconstruction, inequities in the levels of maternal mortality between Indigenous and non-indigenous women remained striking, indicating that the health system was not meeting the needs of Indigenous women (box 1).^9^

Box 1 **Inequities in Indigenous maternal mortality**

In 2000, the maternal mortality ratio for Indigenous women in Guatemala was more than three times that of non-Indigenous women (211 and 70 maternal deaths per 100 000 live births respectively, and an absolute number of 653 maternal deaths overall). This difference fell to 2.1 times that of non-indigenous women in 2007 (163 and 78 maternal deaths per 100 000 live births respectively, and 537 maternal deaths overall), and to 1.75 times by 2015 (139 and 79 maternal deaths per 100 000 live births respectively, and 436 deaths overall).^10^ ^11^ ^12^ One study found that a large portion of ethnic differences in the use of institutional delivery services between Indigenous and non-indigenous women was attributable to Indigenous women not speaking Spanish.^13^ This study and a 2015 health systems assessment for Guatemala^14^ indicate challenges with availability (eg, no qualified health staff at the clinic), accessibility (eg, clinic too far), acceptability (eg, “we cannot give birth the way we want to”), and quality (eg, clinic staff impolite or don’t speak the local language) of services. The findings are also consistent with global evidence on Indigenous women’s use of maternal health services and health outcomes, whereby recommended action by countries includes tackling discrimination; making health centres physically, financially, and culturally accessible; and ensuring equal access to health services.^15^

Service user monitoring generates knowledge and evidence that can be used to advocate for change and improvements. When combined with information on health provider performance and user entitlements, monitoring has been found to lead to better quality and more frequently utilised health services, and ultimately improved health outcomes.^16^ Monitoring by health service users is also an integral part of ensuring the state’s accountability for realising the health and human rights of Indigenous people 17. Monitoring by Indigenous women is therefore key to ensuring the availability, physical and financial accessibility, cultural appropriateness, and quality of health and care services. Since 2008 ALIANMISAR, together with Ministry of Health authorities, has advocated for improved quality, availability, and accessibility of culturally appropriate health services (box 2).

Box 2 **Culturally appropriate health services**

In Guatemala, current legislation defines culturally appropriate health services as those that are:

- Free of discrimination
- Provided bilingually in Spanish and the local Mayan language so the service is accessible to people who communicate in a language other than Spanish
- Focused on the population they serve, with a care model that integrates traditional and modern systems.^18^

A focus on the population served includes the development of norms, practices, and standards to ensure that health services are culturally appropriate and enable Indigenous women to deliver in the most comfortable position for them. For example, vertical birth (giving birth in an upright or squatting position) is a common cultural practice among Indigenous women in Guatemala. This also requires provider training in skills and techniques related to communication, health education, and community engagement to appropriately respond to and respect the culture of Indigenous people.^19^ Health services should also be designed, organised, and implemented in accordance with Indigenous peoples’ values and way of life.

## Joint monitoring by ALIANMISAR and other sectors

ALIANMISAR began monitoring health services for Indigenous women in collaboration with the field offices of the Office of the Human Rights Ombudsman (box 3) in 2010. This was achieved through creation of local networks (REDMISAR; Network of Indigenous women’s organisations for Reproductive Health, Nutrition and Education) and after receipt of technical and funding support from the USAID funded Health and Education Policy Project (HEP+). Monitoring is used to gather evidence about both problems and improvements. Other permanent stakeholders in the monitoring process include the Indigenous men’s network REDHOSEN (Men’s network for Health, Education and Nutrition), municipal government (eg, mayors), and the Ministry of Health (supplementary files 1 and 2).

Box 3 **Office of the Human Rights Ombudsman, Guatemala**

The Office of the Human Rights Ombudsman was created by the National Assembly in 1985 and is responsible for monitoring public sector programmes and performance. The office operates under an agreement between the Office of the United Nations High Commissioner for Human Rights and the Government of Guatemala.^20^ The functions of the ombudsman are to monitor human rights in Guatemala, to provide technical assistance to the government, and to advise state institutions and civil society to enhance the promotion and protection of human rights.

**Fig 1:**
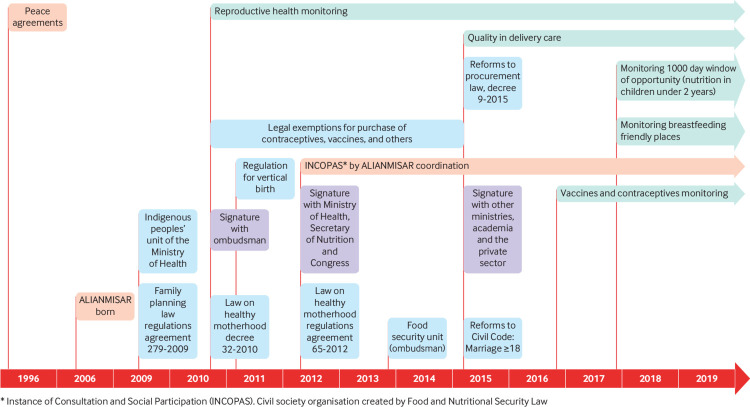
Timeline of ALIANMISAR

ALIANMISAR has developed over time with regard to its main collaborations and the public health topics monitored (fig 1). The range of topics monitored has increased, from reproductive health services in 2010 to include monitoring of nutrition services during the first 1000 days of life. These additions have been driven by political events, such as the health system crisis that led to a reduction in primary healthcare coverage in 2014/15, a reduction in immunisation rates,^14^ ^21^ and the ongoing high rates of chronic malnutrition. For monitoring nutrition services, ALIANMISAR is an elected member of two other entities working on this issue: INCOPAS (the social participation body for food and nutrition security in Guatemala) and CONASAN (the National Food Security and Nutrition Council).

**Fig 2:**
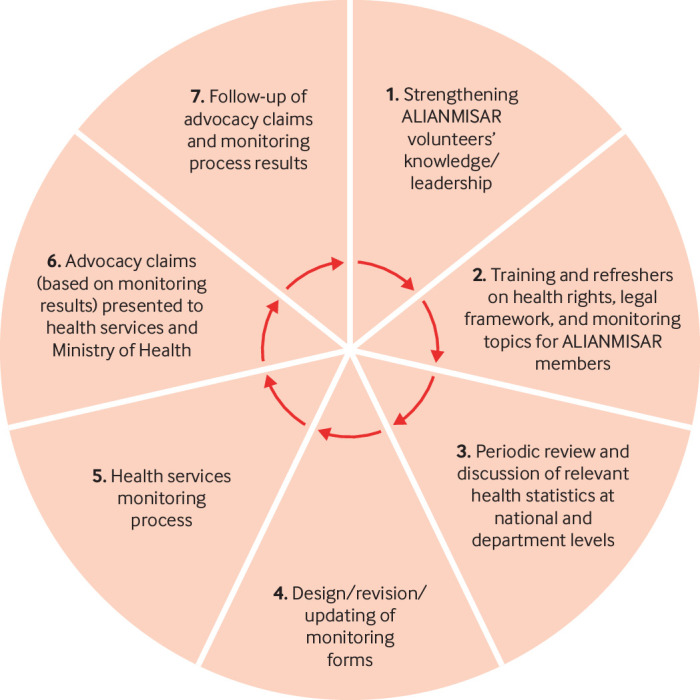
ALIANMISAR’s advocacy and political dialogue process

ALIANMISAR uses an annual monitoring cycle (fig 2) to feed into advocacy efforts: the first four steps include updating knowledge (steps 1 and 2), reviewing data (step 3), and revising forms (step 4). Findings from monitoring, step 5, are used in political dialogue at municipal to national level with the Ministry of Health to bring about improvement in the delivery of high quality and culturally appropriate care and services (step 6).

## From joint monitoring to change

The results of monitoring are used to inform advocacy efforts, including recommendations for political dialogue, and are presented to the Minister for Health annually at a public meeting.^22^ ^23^ Monitoring has contributed to 67 documented improvements in health facilities and services to date^24^—for example, healthcare staff clearly identifying themselves and speaking the local language. ALIANMISAR has also used the results of monitoring to inform important civil society advocacy initiatives for the creation, approval, and implementation of norms, laws, and policies that guarantee access to high quality health services, emphasising cultural appropriateness, and that consider the health needs of Indigenous people (box 4).

Box 4 **Examples of changes to health policy, legislation, services, and infrastructure linked with monitoring and advocacy by ALIANMISAR**

- Creation of the Ministry of Health’s Indigenous Peoples’ Unit in 2009, which is responsible for designing and implementing programmes, policies, and norms to contribute to political and strategic conditions for the right to health of Indigenous people
- Enactment of the Healthy Motherhood Decree of the Congress of the Republic of Guatemala in 2010, aiming to improve the health and quality of life of women and newborns and to strengthen national family planning and reproductive health programmes^25^
- Restoration of the supply of basic drugs, micronutrients, and family planning materials to the health post at Lagunas Cuaches, San Juan Ostuncalco, in October 2012; all key for the provision of preventive health services^24^
- Establishment in March 2017 of a new maternity unit in the hospital in the municipality of Quetzaltenango. This enables pregnant women living in rural areas remote from health services to receive adequate and culturally relevant care before, during, and after delivery^24^

We report an analysis aiming to establish the factors underlying ALIANMISAR’s work which may have contributed to its success in collaborating with other sectors to improve provision of healthcare for Indigenous women in Guatemala. Findings come from a process of document review, key informant interviews, and dialogue with a range of stakeholders at national, departmental, and municipal levels (supplementary file 3).

## Enabling factors for collaboration

We identified five different but complementary factors that enabled ALIANMISAR to successfully collaborate with other sectors and contributed to knowledge and evidence that was used to advocate for changes to health services and care for Indigenous women in Guatemala.

### Legislation and mechanisms for citizen participation

Firstly, having existing legislation and mechanisms that required and supported citizen participation including monitoring has been key. Guatemala’s constitution mandates civil society to hold government accountable, obliging the state to create processes and mechanisms for citizen participation in the governance of health and social sectors.^26^ ^27^ This includes participation in the planning, supervision, execution, and administration of health programmes that are key actions for guaranteeing the right to health.^28^ This meant that when ALIANMISAR was established there was no question about their right to participate in the governance of health services.

This legislative framework has opened the window for the advocacy work of civil society organizations in the protection and promotion of women's development, based on the obligation of the State to provide services and the application of sanctions for violations to its integrity and rights, which is the foundation of the [advocacy] work of ... ALIANMISAR (Andrea Santos, project coordinator, ALIANMISAR) 29

As a signatory to the International Labour Organization 169 Agreement and the UN Declaration on the Rights of Indigenous Peoples^17^ ^30^ the State of Guatemala is obliged to support the right of Indigenous people to participate in decisions that affect them, including development priorities. ALIANMISAR makes use of these mechanisms, including legal and public policy frameworks, to advocate for change and improvements in health services consistent with a human rights based approach to guaranteeing the right to health.

### Existing foundations and networks of ALIANMISAR

Secondly, an existing group of advocates recognised by their Indigenous communities was already in place when ALIANMISAR began. Many Indigenous women who joined ALIANMISAR were already working as volunteers in their communities before its foundation. Most were recognised as credible advocates within their own communities and by other stakeholders. This meant that monitoring findings are seen as reliable with Indigenous communities, as well as with other stakeholders. ALIANMISAR volunteers brought a range of individual skills and experience, including working within the health system as community facilitators, midwives, and health promoters. Most volunteers speak their local Mayan language. Participation by Indigenous women speaking the local language is vital and gives other stakeholders (such as the ombudsman’s office) confidence in the findings. ALIANMISAR’s credibility was further bolstered by its commitment to ongoing follow-up on the results of monitoring to ensure the correct implementation of legislative and public policy mandates (box 4).

Health service stakeholders have also emphasised the value of ALIANMISAR’s credibility for the collaborative work.

When they [the local network] present the findings from the monitoring, we take the opportunity to ask them to talk to community members about using the health services which are open to them, because … communities know them: their members have credibility, and that also helps us to gain their trust (Health sector key informant)

### Resourcing: technical and financial

Thirdly, donor funding of about $160 680 (£125 000; €140 000) annually since 2010 via the HEP+ project has been critical to ALIANMISAR’s work and existing HEP+ technical support. Funding has been used to pay for a national level technical secretary and an additional five technical facilitators to support networks at departmental and municipal levels, and to provide some funds to reimburse ALIANMISAR’s volunteers for travel and related expenses when undertaking monitoring or advocacy activities. USAID funding covers 30 municipalities in departments prioritised by USAID for funding in Guatemala, not all municipalities where ALIANMISAR is active.^3^ ^31^

Training and capacity building in human rights literacy, skills for negotiation, and advocacy with state authorities are important strategies for improving Indigenous people’s participation and advocacy for their own interests.^6^ ALIANMISAR volunteers are trained in the topics/health issues covered by monitoring, as well as in human rights, monitoring, reporting, advocacy, and political dialogue. The HEP+ project coordinator provides training to the HEP+ facilitators and sometimes directly to local network leaders. HEP+ department level technical facilitators assist in compiling, analysing, and presenting the results from monitoring, including prioritisation of findings and development of recommendations for inclusion in reports, presentations, and petitions.

Stakeholders described ALIANMISAR monitors as technically knowledgeable about health rights for Indigenous women and a credible source for other community led organisations, as well as effective advocates, experienced in dealing with the authorities and the media. ALIANMISAR monitors underlined the importance and value of training.

“The training has given me the tools and the confidence to exercise my role, and that has also facilitated successful monitoring” *(ALIANMISAR key informant)*

However, monitoring of health services by ALIANMISAR relies on Indigenous women working as volunteers. Reliance on volunteers was identified by some stakeholders as affecting the sustainability of ALIANMISAR, as volunteers often leave to take up paid employment. Some volunteers, however, noted that the training not only equipped them to undertake monitoring but also built skills that they could use to obtain and/or retain employment.

### Methods used for generating findings for change

Fourthly, the strength of the findings produced and used by ALIANMISAR for advocacy is both an outcome of collaboration with partners, such as the ombudsman and HEP+, and also one of the key enablers of collaboration with the health sector. Findings generated through monitoring fulfil several functions. They serve to identify potential for improvements in healthcare facilities, including service provision. For example, monitoring by ALIANMISAR in 2015 showed that culturally inappropriate practices such as washing women in cold water in health facilities are ongoing and contribute to Indigenous women’s reticence to use those facilities to give birth.^4^ ^32^

After my delivery, they woke me up at 3:00 in the morning so that I could bathe with cold water; they said that if I bathe the doctor would check me, but the doctor never came to check me *(Health services user, Coban Hospital* *32* *)*

The findings also provide a strong foundation underpinning ALIANMISAR’s advocacy for improvements to health policy, protocols, health services, and facilities, resulting in improvements in care (box 4). Consistent and systematic documentation including annual reporting,^22^ ^23^ ^33^ together with the use of media such as photography to document poor conditions of health facilities, and combined with the participatory nature of the monitoring^34^ has been instrumental in persuading health and other stakeholders of the validity and reliability of ALIANMISAR’s findings and the need for proposed changes.

It was from the evidence and results generated by the monitoring exercises, and with their attitude to work, that they gained credibility in the eyes of other actors and improved communications between the different participants in the monitoring process *(Ombudsman KI)*

Sharing the evidence has also improved Indigenous women’s health and human rights literacy.^6^ Using monitoring to identify weaknesses and manage improvements in health services has increased communities’ knowledge of what they are entitled to demand from their health services.

### Shared goals

The fifth enabling factor is shared goals. Multisectoral collaboration is often understood as different sectors acting together to achieve outcomes that cannot be achieved by one sector alone,^35^ usually expressed in terms of shared interests. In this collaboration, however, the shared interests might seem less obvious, because ALIANMISAR has the role of monitoring a key stakeholder, the Ministry of Health. Key informants from health and other sectors reported that collaboration with ALIANMISAR has helped them to do their job better, achieve their goals, and, crucially, improve their own credibility. For example, in one health service, collaboration led to a change in communication style: the respectful behaviour by staff that had long been called for was finally achieved when it was formally recommended after an audit by ALIANMISAR. In another health service, a key informant said that the monitoring report produced by ALIANMISAR is a tool that can be used for follow-up with the Ministry of Health not only by ALIANMISAR but also by the health services. It provides them with documentation of the need for local resource allocation so that services and changes to facilities can be made to ensure culturally acceptable health services are available.

In terms of other sectors, the collaboration means that the local field officers of the ombudsman accompany ALIANMISAR monitors and can cover a wider geographical area in monitoring the right to health because the number of areas monitored is greater than they would cover alone. Furthermore, monitors from the ombudsman’s office may not speak the language of the region where they work, so collaboration with women who speak the local language helps them to reach service users more effectively.

However, it has taken time for collaboration to be recognised as mutually beneficial. One stakeholder from the health sector described how they initially thought the purpose of monitoring was to audit the ministry but came to appreciate that its real purpose was to enable and support the health sector to do their job by pointing to the improvements in health services needed to achieve the goal of wellbeing for people (box 5). Some stakeholders identified frequent changes of administration and staff at all levels in Guatemala as a challenge for collaboration, since these changes often require local networks to rebuild relationships from the beginning.

Box 5 **Constructive dialogue with health professionals about culturally appropriate childbirth and delivery methods**

To make childbirth practices and delivery methods in indigenous contexts more culturally appropriate, ALIANMISAR approached the medical and nursing schools of the State University, advocating that students be trained so that women can deliver in the position they find most comfortable. For example, vertical birth is a common cultural practice for Indigenous women. However, senior staff in the medical faculty were initially resistant. Volunteers had attended an exchange with Peru about their childbirth practices and ALIANMISAR subsequently returned to the university to discuss their findings with the medical faculty. Through this exchange, the university was motivated to implement a series of short training sessions on the topic to raise awareness among students completing supervised professional training in health services and plans to include these traditional methods in the school’s training curriculum.

We found that these five factors were key both to the collaboration and its success in advocating for change to health services for Indigenous women. ALIANMISAR’s continuous presence in the political space, using existing legal frameworks, the reliability of findings from monitoring, and technical assistance to the health sector have made them a legitimate, credible, and trustworthy partner. This has increased collaborators’ willingness to respond to advocacy for change as seen by improvements to health policy, infrastructure, and services.^36^

## Challenges and limitations

Stakeholders indicated an ongoing need for ALIANMISAR’s work and for its expansion. However, the review process also identified challenges for the collaboration and for ALIANMISAR overall. Firstly, questions surround sustainability and equity of resourcing: ALIANMISAR is funded by short term grants from donors. Resourcing for ALIANMISAR’s work affects monitoring in a number of ways. Health services monitoring takes place in only six of the 22 departments, and other departments with Indigenous populations may be being missed. Additionally, USAID funding provides for 30 municipalities and does not cover all those where ALIANMISAR is present. In municipalities without USAID funding, ALIANMISAR still conducts annual monitoring exercises, sometimes with financial support from other stakeholders such as the local municipality and the ombudsman.

Secondly, while the voluntary nature of Indigenous women’s participation in ALIANMISAR gives credibility to their work, it also presents challenges, including a high turnover of volunteers. The collaboration and its successes is also dependent on unpaid work by Indigenous women, which is inconsistent with the principles of equity and gender equity.^37^ ^38^

These challenges do not detract from the collaboration’s success nor from ALIANMISAR’s achievements, but they do show that a strategic review of ALIANMISAR’s collaborative work with the Ministry of Health, the ombudsman’s office, and other stakeholders in improving the health and wellbeing of Guatemala’s Indigenous women and their communities would be timely. As well as exploring how to fund ALIANMISAR in the long term it needs to include an evaluation of how it works, what it works on, and the outcomes and impacts for Indigenous women and communities.

## Conclusion

Despite a commitment to formal mechanisms for civil society participation in governance post-1996 in Guatemala, Indigenous people, particularly Indigenous women, were not participating fully in those processes to effectively advocate for their interests and rights.^1^ ^6^ ^7^ The creation of ALIANMISAR as an organisation run by Indigenous women for Indigenous women was an important response to this gap, both enabled by and resulting in more effective use of these participatory mechanisms.

We highlight the experience of, and challenges involved in, community led, multisectoral collaboration for improving the availability, accessibility, cultural acceptability, and quality of health services for Indigenous women. This experience shows what can be achieved in a low resource setting by an existing network of respected community volunteer advocates, with additional resources, capacity building, and a long term commitment to improving the health system. To produce long term improvements in Indigenous women’s lives, it is essential to continue building on ALIANMISAR’s work and successes in a sustainable and equitable way. The findings from the review process will therefore be used to inform future efforts by ALIANMISAR.

**Key messages**

- ALIANMISAR monitors a range of public health services, in collaboration with other community based organisations, the Ministry of Health, the Ombudsman for Human Rights, and international partners, to generate evidence for improvements to the quality and cultural acceptability of health services for Indigenous women
- Previous work by Indigenous women as advocates in their own communities aided collaboration with ALIANMISAR, bringing additional technical and financial resources to enable further advocacy
- ALIANMISAR’s methods and its presence in the political space for many years makes it a legitimate, credible, and trustworthy partner, facilitating of health and other sectors to respond to its advocacy claims
- A strategic review is needed to determine how to fund and structure ALIANMISAR in future to build on existing gains in a sustainable and equitable way

## Web Extra

Extra material supplied by the author

supplement 1: Stakeholder information

supplement 2: Monitoring of health services by Indigenous women for Indigenous women

supplement 3: Methods for the case study

## Notes

See www.bmj.com/multisectoral-collaboration for other articles in the series.

## Notes

Contributors and sources:SJS was supervisory author; DU, SJS, and CNV conceived the article outline; SJS and DU drafted and revised the first version with CNV and RGM; and SX and SP provided feedback and key inputs on drafts. For the case study review SJS and CNV wrote the draft working paper with inputs from other authors; CNV and RGM undertook key informant interviews; and CNV, RGM, SJS, and DU drafted an initial summary of findings used for consultation. All authors contributed intellectual content and approved the final version of this article for submission, with CNV serving as guarantor of the article.

Provenance and peer review: Commissioned; externally peer reviewed.
