# Supplementary material for: Making the health system work by and for Indigenous women in Guatemala: a community led multisectoral collaboration
Source: BMJ. 2018 Dec 7;363:k4677. doi: 10.1136/bmj.k4677 (PMC6284262; doi:10.1136/bmj.k4677)
Supplement: Supplementary file 1 — supplement 1: Stakeholder information [file niec47381.ww1.pdf]

SUPPLEMENTARY INFORMATION/APPENDICES

Supplement 1: Stakeholder Table

STAKEHOLDERS	ROLE
**CIVIL SOCIETY**	
**REDMISAR** (Network of Indigenous women's organizations for Reproductive Health, Nutrition and Education)	Eight local networks at the departmental level that form ALIANMISAR. Their members carry out the monitoring exercises and participate in advocacy work of ALIANMISAR. The nature and thematic interests of organizations forming REDMISAR's are diverse and include grassroots youth organizations, agricultural producers, women weavers, traditional midwives, among others, all of which are also committed in one way or another to the health rights of Indigenous populations and reproductive health in particular.
**REDHOSEN** (Men's network for Health, Education, and Nutrition)	A smaller and newer network similar to ALIANMISAR, formed by men that that seek to work with other men in promoting responsible masculinity issues. Supports ALIANMISAR in conducting the monitoring of health services as well as in lobbying and advocacy efforts
**OSAR** (Reproductive health observatory)	The observatory's members include academic institutions. The observatory is supported by Congress and has representation at the departmental level. Its work converges with ALIANMISAR's efforts to monitor and supervise the implementation of public policies related to reproductive health and in two departments (Quetzaltenango and Quiché) local OSAR members are involved in the monitoring.
**GOVERNMENT OF GUATEMALA: LEGISLATIVE**	
**Human Rights Ombudsman**	Accompanies and validates monitoring exercises which adds credibility, guarantees access to health services and the legitimacy of the monitoring,

STAKEHOLDERS	ROLE
	accompanies presentation of monitoring results with MOH officials, follows-up the implementation of the demands presented to the MOH based on monitoring results, training in health rights. ALIANMISAR signed a working agreement with the Ombudsman first in 2010, and this has been ratified to date three times.
**National Congress Commissions** (Women's Health Commission, Consejo Parlamentario de Salud (COPAS) and the Commission of Indigenous People)	Have collaborated with ALIANMISAR since 2011, signing a letter of understanding that enables different congress commissions to collaborate in ALIANMISAR's advocacy efforts, serving as mediators and following up with government entities such as the MOH to guarantee and follow up the demands resulting from the monitoring of health services.
**GOVERNMENT OF GUATEMALA: EXECUTIVE**	
**Food and Nutritional Security Secretariat (SESAN)**	SESAN is the government office responsible for the coordination, integration and monitoring of Food Security interventions implemented by the government, public sector, civil society and international cooperation agencies. The relationship between SESAN and ALIANMISAR was born in 2012 as an independent, complementary effort to monitor the essential nutrition actions for the 1000-day window of opportunity implemented by health services.
**Ministry of Health and Social Assistance (MSPAS)**	Signed working agreement in June 2009, which has been ratified twice, committing to working with Indigenous people and to incorporating the civil society in the auditing of the health of Indigenous women. It is the recipient of ALIANMISAR's monitoring of health services and in charge of making the necessary improvements in the quality of health services to respond to the demands resulting from the

STAKEHOLDERS	ROLE
	monitoring. The collaborative work is done through the health areas and districts and the Indigenous Health Unit.
**Municipalities**	The municipal governments facilitate community participation and intersectoral collaboration, finance important infrastructure projects to support the network of health services, specifically health posts and centres, and sometimes contribute staff and resources for the implementation of local activities including the monitoring exercises. In some cases, in response to monitoring by ALIANMISAR, municipalities have supported the hiring of health workers including nurses and auxiliary nurses to strengthen the provision of primary care. Depending on the person in power, municipalities have also supported ALIANMISAR in the advocacy efforts to follow up on improvements needed in the health services.
**PRIVATE SECTOR**	
**Alliance for nutrition**	Brings together entities from the private sector that advocate to improve chronic malnutrition and influence opinion and public policy on food security and the correct implementation of the essential nutrition actions in the country. Worked with ALIANMISAR in 2017 to monitor vaccination coverage.
**DONORS/TECHNICAL ASSISTANCE**	
**USAID Guatemala Mission (Health and Education Unit)**	ALIANMISAR's main donor, supports training to strengthen advocacy efforts and contributes to improve and optimize monitoring exercises, through its health and education projects.
**USAID/HEP+ (Health Policy Initiative, Health and**	Funds training of volunteers, fosters leadership and provides technical assistance on advocacy and

STAKEHOLDERS	ROLE
**Education Policy Project and HEP+)**	monitoring processes. Has provided technical assistance and accompanied ALIANMISAR from its origins, in addition to be its main source of financing (through USAID funds). The HEP+ support was also instrumental in training processes to strengthen capacities and abilities of the leaders, technical support in strategic and operational planning and monitoring, presentation of results and monitoring the implementation of the actions for improvement.
**USAID/FANTA III** (Food and Nutritional Technical Assistance Project)	Financed training of some sessions for ALIANMISAR leaders, including a Certificate Course on nutrition during the first 1000 days of life, and additional trainings on malnutrition, monitoring, micronutrients, legislation, and others. FANTA's technical assistance was also valuable to strengthen the collaborative work with the Ombudsman's Office, as well as in the review of the monitoring results presentations for Congress.
**USAID/MCSP Maternal and Child Survival Program**	Provided technical assistance to develop a mobile application to advance and modernize the monitoring of health services to allow for more automated form to collect, analyze and use data.
