# Supplementary material for: Making the health system work by and for Indigenous women in Guatemala: a community led multisectoral collaboration
Source: BMJ. 2018 Dec 7;363:k4677. doi: 10.1136/bmj.k4677 (PMC6284262; doi:10.1136/bmj.k4677)
Supplement: Supplementary file 2 — supplement 2: Monitoring of health services by Indigenous women for Indigenous women [file niec47381.ww2.pdf]

**Supplement 2: The monitoring of health services by Indigenous women for Indigenous women**

The ALIANMISAR coordinators at departmental level work with local monitors to undertake monitoring 1-2 times a year in each of the relevant and prioritized municipalities. They organize the visits with the ombudsman's office and representatives at the local level networks for ALIANMISAR and community-based organizations. The local health service is not aware of the monitoring dates and must be prepared for the monitor's attendance. Stakeholder interviews indicated that the process is well known and regarded at local level: "The mayor already knows us and attends, as in the services, we have no problems in entering." (Civil Society KI) The monitors, observe the health services being delivered, identify if the required medical supplies and or equipment are available e.g. vaccines and interview Indigenous women about the quality of service. The exercise usually also involves taking photos and a mobile application has been developed for monitors to use in collecting data. Monitoring takes between 0.5-1 day for each site, depending on how many monitors attend and each monitoring exercise can last between 1 and 2 months at a time. The tabulation of data at local level takes approximately 7 days. Data is then tabulated at the departmental level and takes 15 to 20 days. This process includes the analysis and development recommendations for change. The results from the departments are consolidated into a report and presented to the Minister for Health annually as part of an ongoing dialogue. The media attend the official launch of ALIANMISAR's annual report to the Minister of Health.
