# Supplementary material for: Making the health system work by and for Indigenous women in Guatemala: a community led multisectoral collaboration
Source: BMJ. 2018 Dec 7;363:k4677. doi: 10.1136/bmj.k4677 (PMC6284262; doi:10.1136/bmj.k4677)
Supplement: Supplementary file 3 — supplement 3: Methods for the case study [file niec47381.ww3.pdf]

**Supplement 3. Methods for ALIANMISAR case study**

This case study was developed following the PMNCH case study guide and multi-stakeholder dialogues guide developed through the 2014 Success Factors studies process.[1],[2] Data was collected and analysed in April-July 2018 and included: 1) document review; 2) key informant interviews (May and June 2018); and 3) a multi-stakeholder review.

1. Document review. Primary and secondary documentation and data were identified by ALIANMISAR and HEP+. This was further supplemented by searches by the review team. Materials reviewed included strategy, planning and monitoring reports, presentations, partner letters of understanding, newspaper publications, website information, and peer-reviewed academic publications both in English and Spanish.

2. Key informant (KI) interviews. Individual KI interviews (n=29) and one group interview (n=12) with representatives of various stakeholder entities (N=9) were conducted at the national, departmental, and municipal levels to ensure a diversity of perspectives consistent with the WHO multi-stakeholder process.

Nine of the KI interviews were conducted during health services monitoring exercises in three municipalities (Concepción Chiquirichapa, Chajul, and Chichicastenango). The decision to carry out interviews in the three selected municipalities was, first, due to their diversity in terms of infrastructural improvements achieved by REDMISAR monitoring and the resources invested in monitoring at this level. Secondly, site selection was also driven by considerations of efficiency and convenience, since the monitoring exercises in those municipalities coincided with the period of our own data collection, which was time and financially bound. The limited number and non-random selection of municipal level research sites represents a limitation of this case study. However, significant diversity both in terms of inputs and outcomes between the selected municipalities suggests sufficiently high external validity of the findings.

---

[1] Partnership for Maternal Newborn and Child Health (PMNCH). What works and why? Success Factors for collaborating across sectors for improved women's, children's and adolescents' health. [Internet]. WHO; 2018 [cited 2018 Jul 19]. Available from: http://www.who.int/pmnch/knowledge/case-studies/en/

[2] Frost L, Hinton R, Pratt B-A, Murray J, Arscott-Mills S, Jack S, et al. Using multistakeholder dialogues to assess policies, programmes and progress for women's, children's and adolescents' health. Bulletin of the World Health Organization. 2016;94:393–5.

An initial list of key informants that played some role in supporting the monitoring of health services was proposed by ALIANMISAR. Snowball sampling was used to identify additional key informants.

Questions for the interview guide were adapted from the WHO case study guide and covered the following topics: motivation to collaborate; identification of the stakeholder's role; identification of factors that facilitated the success; and the main challenges of the multisectoral work with ALIANMISAR. The interviews were conducted in Spanish. Participation was voluntary, and informants gave oral consent to recording of the interviews. To ensure confidentiality, all KI interviews and stakeholder responses have been de-identified.

Digitally-recorded interviews and field notes were analyzed employing the method of directed content analysis. The analysis proceeded both deductively, i.e. relying on the categories from the PMNCH case study guide, and inductively, which led to creation of new categories emerging from the data.

3. Multi-stakeholder review process. Validity and reliability of the findings from the document review and KI interviews were assessed with multiple informants from diverse settings in two consultation exercises. The consultations were done using a short summative paper on key findings and conclusions of the case study. The first consultation was conducted with local representatives (n=28) of various stakeholder entities (N=7) in five departments. The second one was done with central level representatives (n=3) of stakeholder entities (N=3). Stakeholder feedback on the document has been reflected in the working report and this article.
